# Development of a Duplex TaqMan-MGB qPCR Assay for Differential Detection of Chinese Epidemic Lumpy Skin Disease Virus Strains and Goatpox Virus

**DOI:** 10.3390/ani16162561

**Published:** 2026-08-17

**Authors:** Siyang Mu, Majiancai Bai, Xiaohu Zhang, Manyang Yu, Yaozhong Lu, Mengen Xu, Haofang Yuan, Chuxian Quan, Zhun Yi, Lan He, Yan Li, Jiakui Li

**Affiliations:** 1 College of Veterinary Medicine, Huazhong Agricultural University, Wuhan 430070, China; msy1011@webmail.hzau.edu.cn (S.M.);; 2 Agriculture and Animal Husbandry Comprehensive Service Center, Longzi 856600, China; xzzhmyz@163.com; 3 Abz Academy of Agricultural Sciences, Aba 624000, China; 4 Hubei Jiangxia Laboratory, Wuhan 430200, China

**Keywords:** duplex TaqMan-MGB qPCR, LSDV, GTPV, yaks, the Qinghai–Tibet Plateau

## Abstract

Lumpy skin disease (LSD) is a transboundary viral disease of cattle, including Asian water buffalo and yaks, and certain wild ruminants (e.g., African buffalo, giraffe, wildebeest, eland, and Arabian oryx). It is caused by lumpy skin disease virus (LSDV), a member of the genus *Capripoxvirus* (family *Poxviridae*) together with goatpox virus (GTPV) and sheeppox virus (SPPV). High nucleotide identity and serological cross-reactivity among these viruses hinder differential diagnosis. The aim of this study was to develop a Duplex TaqMan-MGB qPCR Assay for Differential Detection of Chinese Epidemic Lumpy Skin Disease Virus Strains and Goatpox Virus. We developed a duplex TaqMan-MGB real-time PCR (qPCR) assay targeting the LSDV *GPCR* and GTPV *RPO30* loci. Virus-specific primers and MGB probes were designed, and the reaction was optimized for single-tube, two-target detection. The assay showed no cross-amplification, limits of detection of 1 × 10^1^ copies/μL (LSDV) and 1 × 10^1^ copies/μL (GTPV), and coefficients of variation < 1%. The assay was applied to 175 yak-derived field specimens from Qinghai–Tibet Plateau, of which 16 and 36 were positive for LSDV and GTPV, respectively. LSDV- and GTPV-positive samples showed specific amplification in the FAM and VIC channels, respectively. The duplex format enables concurrent detection and unambiguous differentiation of LSDV and GTPV and is compatible with high-throughput screening. This sensitive, specific, and reproducible assay supports surveillance and control of LSD in endemic and at-risk regions.

## Simple Summary

Lumpy skin disease is an important viral disease of cattle and yaks that is associated with fever, skin nodules, reduced productivity, and economic losses. In China, goatpox virus vaccines have been used to help control this disease, but vaccine-related reactions may look similar to the clinical signs of natural infections. In addition, lumpy skin disease virus and goatpox virus are closely related, making them difficult to distinguish using clinical signs or routine serological tests. In this study, we developed a duplex TaqMan-MGB real-time PCR assay to detect Chinese epidemic lumpy skin disease virus strains and goatpox virus in a single reaction. The assay was sensitive, specific, and reproducible, and it showed no cross-reaction with other tested pathogens. When applied to 175 field samples from yaks in the Qinghai–Tibet Plateau region, the assay successfully identified positive samples for both viruses. This method shows potential as a tool for molecular detection and surveillance of LSDV and GTPV, especially in regions where cattle and yak farming are important.

## 1. Introduction

Lumpy skin disease (LSD) is an acute, subacute, or chronic infectious disease of cattle caused by lumpy skin disease virus (LSDV). LSDV belongs to the genus *Capripoxvirus* within the family *Poxviridae*, together with goatpox virus (GTPV) and sheeppox virus (SPPV) [1]. These three *Capripoxvirus* share high nucleotide sequence identity (>96%) and induce cross-reactive antibodies, rendering serology unreliable for differentiating infections. In LSDV-affected herds, morbidity typically ranges from 5–45%, and mortality is around 10% [2]. Early clinical signs include rhinitis and conjunctivitis with purulent nasal and ocular discharge; as disease progresses, keratitis may develop, accompanied by high fever (>41 °C). Subsequently, firm, round, raised nodules appear on the skin and mucosa throughout the body, generally 2–7 cm in diameter and are painful on palpation [3].

LSD was first identified in Zambia in 1929 and, over subsequent decades, spread gradually northward to involve nearly the entire African continent. Following the first report in Türkiye in 2013, the disease expanded across parts of Europe and Asia, posing a growing threat to cattle production [4,5]. In China, LSD was first reported in Xinjiang in 2019; since then, official notifications have documented outbreaks in 14 provinces/regions, including Fujian, Jiangxi, Guangdong, Anhui, Zhejiang, Sichuan, Yunnan, Hong Kong, Shandong, Inner Mongolia, and Taiwan [6,7,8,9,10,11]. In October 2021, the first case was identified in yaks and yak–cattle hybrids, indicating spread into high-altitude pastoral areas previously considered at lower risk [8]. A substantial body of experimental and field evidence indicates that LSDV is transmitted mainly by blood-feeding arthropods—including mosquitoes, biting midges (*Culicoides* spp.), and hard (ixodid) ticks—which explains rapid dissemination in vector-abundant environments [12]. In yaks, LSD causes fever and nodular lesions of the skin and mucous membranes and can result in high mortality [8]. Collectively, these consequences directly and negatively impact the sustainability and economic performance of yak husbandry on the Qinghai–Tibet Plateau [13].

In China, an attenuated live GTPV vaccine is officially recommended for LSD prevention [14]. However, post-vaccination cutaneous reactions—typically sparse nodules (≈5–20 mm) over the back, flanks, and shoulders—can be clinically indistinguishable from infection with currently circulating LSDV strains, indicating that clinical presentation alone lacks discriminatory value [15]. In China, currently circulating LSDV strains belong predominantly to a recombinant lineage that differs genetically from classical foreign LSDV strains. In addition, live-attenuated goatpox virus vaccines are widely used for LSD prevention, making molecular discrimination between Chinese epidemic LSDV strains and GTPV particularly important for outbreak investigation, vaccine monitoring, and epidemiological surveillance. To distinguish infections caused by Chinese circulating LSDV strains from post-vaccination reactions to the attenuated GTPV vaccine, and to differentiate vaccine-like recombinant LSDV from GTPV, the aim of this study was to develop and evaluate a duplex TaqMan-MGB qPCR assay targeting the LSDV GPCR and GTPV *RPO30* gene regions for the differential detection of Chinese epidemic LSDV strains and GTPV.

## 2. Materials and Methods

### 2.1. Viruses, Vaccine Strains, and Clinical Samples

Gene fragments corresponding to the *GPCR* region of a Chinese circulating LSDV strain and the *RPO30* region of GTPV were synthesized by Sangon Biotech (Shanghai) Co., Ltd., Shanghai, China. The attenuated goatpox virus vaccine strain AV41, the live contagious ecthyma (orf) vaccine (HCE strain; Shandong Huahong Bioengineering Co., Ltd., Binzhou, China), and the live peste des petits ruminants (PPR) vaccine (Xinjiang Tiankang Biological Co., Ltd., Urumqi, China.) were used as reference materials to evaluate analytical specificity. In addition, the following pathogens were maintained in our laboratory and included in the specificity panel: LSDV/China/SiC/2021, rotavirus, bovine viral diarrhea virus (BVDV), bovine coronavirus (BCoV), bovine herpesvirus 1 (BHV-1; infectious bovine rhinotracheitis virus), and the bacterial strains *Staphylococcus aureus*, *Salmonella* spp., and *Escherichia coli*.

### 2.2. Primers, and Probes

Sequences of 33 *Capripoxvirus* strains (LSDV, GTPV, and SPPV) available in GenBank were retrieved, and multiple sequence alignments (MSAs) of the *GPCR* and *RPO30* gene regions were generated using MEGA v11 (Arizona State University, Tempe, AZ, USA), with specific details listed in Table 1. Primers and TaqMan-MGB probes were designed using Primer Express v3.0.1 (Applied Biosystems, Foster City, CA, USA) and Primer Premier v5.0 (PREMIER Biosoft International, Palo Alto, CA, USA) to (i) specifically detect Chinese epidemic LSDV strains at *GPCR* and (ii) detect GTPV at *RPO30* while avoiding cross-reactivity with LSDV/SPPV. In silico specificity was assessed using BLASTn (National Center for Biotechnology Information, Bethesda, MD, USA) against the NCBI nt database. Probes were labeled with FAM (LSDV-GPCR) and VIC (GTPV-RPO30). All oligonucleotides were synthesized by Sangon Biotech (Shanghai) Co., Ltd., Shanghai, China. Detailed data is presented in Table 2.

### 2.3. Construction of Plasmid Standards and Copy Number Calculation

Synthetic fragments covering the target regions were cloned into pUC57 to generate plasmid standards (pUC57-LSDV and pUC57-GTPV) and sequence-verified by Sangon Biotech (Shanghai) Co., Ltd., Shanghai, China. Plasmid concentration (ng/μL) was measured using Nanodrop 2000 spectrophotometer (Thermo Fisher Scientific, Waltham, MA, USA). Copy number was calculated as

copy number/μL = (6.02 × 10^23^ × concentration (ng/μL) × 10^−9^)/(plasmid length (bp) × 660).

Ten-fold serial dilutions were prepared from 1 × 10^9^ to 1 × 10^0^ copies/μL in nuclease-free water.

### 2.4. Manufacturer-Recommended Duplex TaqMan-MGB qPCR Conditions

Initial reactions were tested using the manufacturer-recommended concentrations. Duplex reactions were performed in 20 μL containing 10 μL AceQ Universal U+ Probe Master Mix V2 (Vazyme Biotech Co., Ltd., Nanjing, China), primers at final concentration, 0.2 μM, probes at final concentration, 0.2 μM, and 2 μL template DNA. Cycling was conducted on a QuantGene 9600 Real-Time PCR Analyzer (Hangzhou Bioer Technology Co., Ltd., Hangzhou, China) under the following conditions: 37 °C incubation for 2 min for UNG/UDG-mediated carryover contamination control, followed by 95 °C for 5 min for initial denaturation and enzyme activation, and then 45 cycles of 95 °C for 10 s, 58 °C for 30 s (fluorescence acquisition), and 72 °C for 30 s. Fluorescence was collected in the FAM and VIC channels. Manufacturer-recommended conditions were used as baseline settings; the final optimized duplex reaction composition is described in Section 3.1.

### 2.5. Optimization Strategy

Primer and probe concentrations and annealing temperature were optimized using mixed plasmid templates (1 × 10^6^ copies/μL each) to minimize Ct values while maintaining high fluorescence amplitude and absence of nonspecific amplification. The tested ranges were primers 0.10–0.20 μM, probes 0.10–0.20 μM, and annealing temperatures 52–58 °C.

### 2.6. Standard Curves, Linearity, and Duplex TaqMan-MGB qPCR Efficiency

Standard curves for each target were generated using ten-fold serial dilutions (1 × 10^9^–1 × 10^0^ copies/μL) tested in triplicate. Ct values were regressed against log_10_(copy number) to determine slope and R^2^. Amplification efficiency was calculated as E = (10^(−1/slope)^ − 1) × 100%.

### 2.7. Evaluation of Specificity, Sensitivity, and Reproducibility

To evaluate the analytical specificity of the assay, the goatpox virus vaccine strain AV41 and LSDV/China/SiC/2021 were used as positive controls. In parallel, for RNA viruses including PPRV, BRV, BVDV, and BCoV, viral RNA was first reverse-transcribed into cDNA using the HiScript IV All-in-One Ultra RT SuperMix for qPCR (Vazyme Biotech Co., Ltd., Nanjing, China) according to the manufacturer’s instructions. The resulting cDNA, along with the extracted DNA from DNA viruses (orf virus and BHV-1) and bacteria (*Staphylococcus aureus*, *Salmonella* spp., and *Escherichia coli*)*,* were subjected to amplification under the optimized duplex TaqMan-MGB qPCR conditions to assess potential cross-reactivity. Nuclease-free water served as the no-template control (NTC).

To assess analytical sensitivity, ten-fold serial dilutions (1 × 10^0^ to 1 × 10^9^ copies/μL) of mixed plasmid standards containing the LSDV and GTPV targets were prepared. Each dilution was tested by duplex TaqMan-MGB qPCR under the optimized conditions. For each target, LOD was defined as the lowest concentration with 100% positive detection in 8 independent replicates.

Reproducibility was assessed across three independent runs. In each run, plasmid standards at 1 × 10^6^, 1 × 10^5^, and 1 × 10^4^ copies/μL were tested in triplicate. Variability was quantified using the coefficient of variation (CV), defined as (standard deviation/mean) × 100%. Intra-assay CVs were calculated from the triplicate measurements within a single run, whereas inter-assay CVs were derived from the results across the three runs, providing an objective evaluation of assay reproducibility.

### 2.8. Clinical Specimen Testing

Approximately 1 g of skin tissue was homogenized in 1 mL phosphate-buffered saline (PBS) using an Automatic Sample Fast Grinder (Shanghai Jingxin Industrial Development Co., Ltd., Shanghai, China). The homogenate, along with other samples, was centrifuged at 9000 rpm for 3 min at 4 °C. Blood samples were subjected directly to nucleic acid extraction without pretreatment. Viral DNA was extracted from 400 μL of each sample using the VAMNE Magnetic Pathogen DNA/RNA Kit (Prepackaged) (Vazyme Biotech Co., Ltd., Nanjing, China) and an automated nucleic acid extraction system (Vazyme Biotech Co., Ltd., Nanjing, China) according to the manufacturer’s instructions.

Using the duplex TaqMan-MGB qPCR assay developed in this study for detecting GTPV and Chinese circulating LSDV strains, we tested clinical specimens, LSDV Neethling *GPCR* synthetic gene constructs, and nucleic acids extracted from the attenuated goatpox vaccine strain AV41. The clinical specimens included EDTA-anticoagulated whole-blood, muscle, and skin samples collected from individual yaks between 2011 and 2024 in Tibet (Xizang), Sichuan, Qinghai, and Gansu provinces. Each sample represented a single clinical specimen collected from one individual yak, and only one specimen type was collected from each animal. All specimens were stored at −80 °C until analysis. A total of 175 field specimens (175 individual yaks) were included, comprising 102 specimens collected before 2019 and 73 specimens collected during 2019–2024. Pre-2019 specimens were included as historical samples to assess assay specificity and background circulation prior to the first reported LSDV outbreak in China. Clinical specimens were considered positive when a typical sigmoidal amplification curve was observed and the Ct value was ≤35. Clinical specimens with Ct > 35 or no amplification were considered negative. No repeat testing was performed. Sample interpretation was based on the predefined positivity criterion (Ct ≤ 35) and amplification curve characteristics, and no inconclusive or invalid results were observed in this study.

## 3. Results

### 3.1. Optimization of Duplex TaqMan-MGB qPCR Conditions

**Figure 1:**
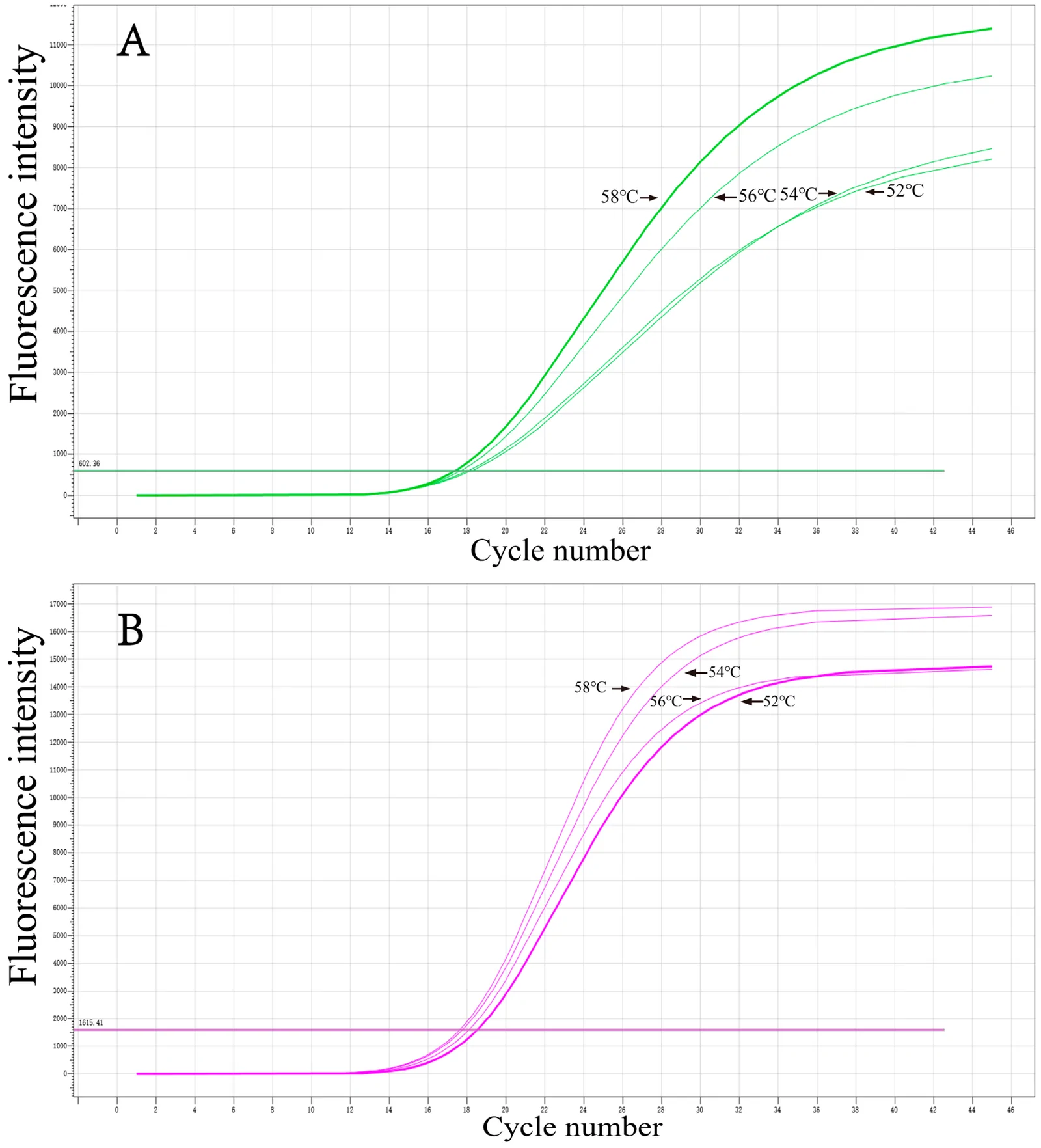
Optimization of annealing temperature for the duplex TaqMan-MGB qPCR assay. Amplification was performed with an initial incubation at 37 °C for 2 min, followed by denaturation and enzyme activation at 95 °C for 5 min, and 45 cycles of 95 °C for 10 s, 52–58 °C for 30 s (fluorescence acquisition), and 72 °C for 30 s. (**A**) Amplification curves of the LSDV *GPCR* target. (**B**) Amplification curves of the GTPV *RPO30* target. The horizontal line represents the fluorescence threshold used for Ct determination.

To optimize the duplex TaqMan-MGB qPCR assay, we evaluated annealing temperatures ranging from 52 to 58 °C and primer/probe concentrations from 0.10 to 0.20 µM. The optimal condition was selected based on target-specific amplification, the lowest Ct values, and the highest fluorescence intensity. Among the tested conditions, 58 °C produced the lowest Ct values and the strongest amplification signals and was therefore selected as the optimal annealing temperature (Figure 1). Optimization of primer and probe concentrations showed that 0.3 μL of GTPV primers, 0.4 μL of LSDV primers, and 0.4 μL of probes for both targets yielded the best amplification performance, with the lowest Ct values among the tested conditions (Table 3 and Table 4). Under this condition, the final 20 µL duplex reaction contained 10.0 µL AceQ Universal U+ Probe Master Mix V2, 0.3 µL each of GTPV forward and reverse primers, 0.4 µL each of LSDV forward and reverse primers, and 0.4 µL each of GTPV and LSDV probes, 2.0 µL DNA template, and ddH_2_O added to a final volume of 20.0 µL.

### 3.2. Establishment of Standard Curves

**Figure 2:**
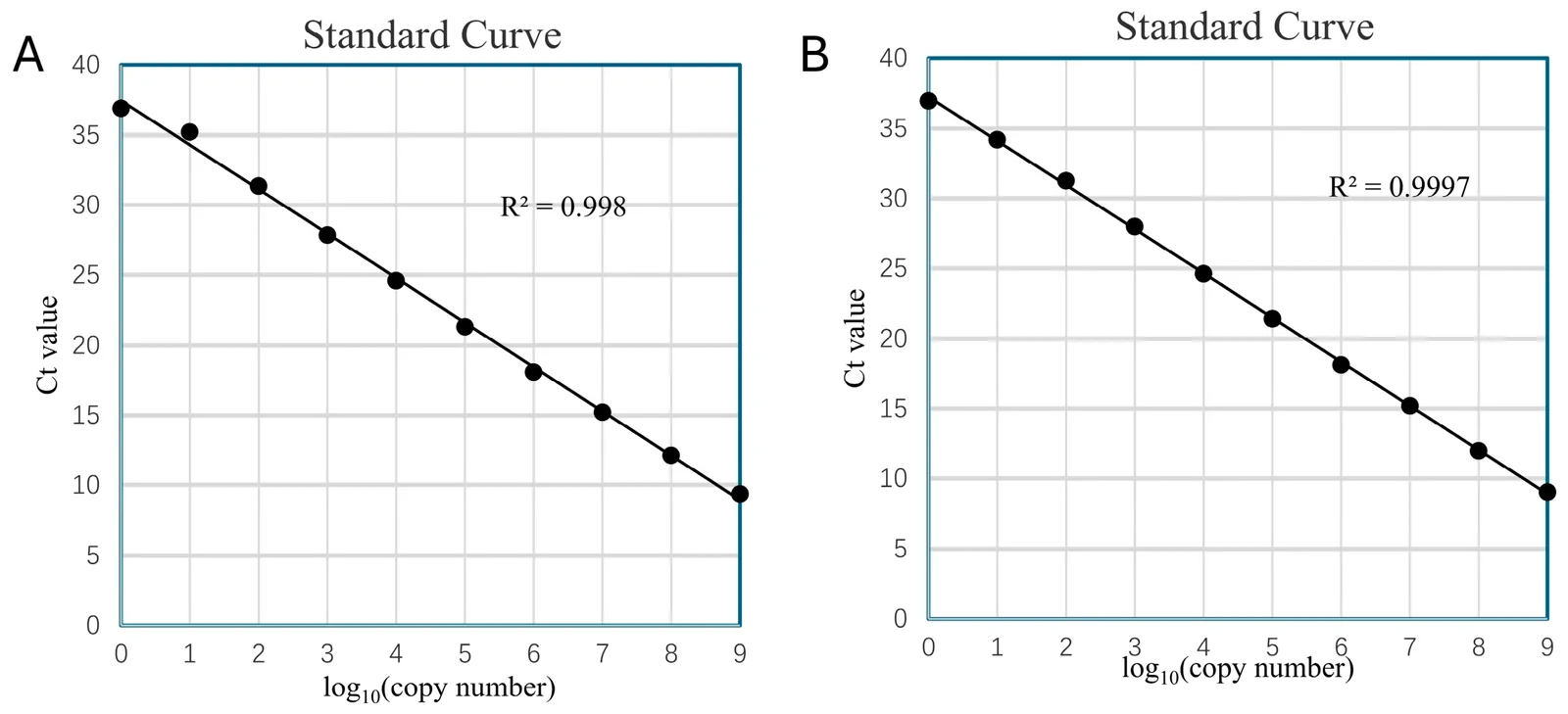
Standard curves of the Duplex TaqMan-MGB qPCR assay. (**A**) Standard curve for LSDV. (**B**) Standard curve for GTPV. Standard curves were generated using ten-fold serial dilutions of plasmid DNA ranging from 1 × 10^9^ to 1 × 10^0^ copies/μL. The R^2^ values are shown in the corresponding panels.

Plasmid standards for LSDV and GTPV were serially diluted from 1.0 × 10^9^ to 1.0 × 10^0^ copies/μL and used as templates to construct standard curves under the optimized duplex TaqMan-MGB qPCR conditions. Linear regression analysis demonstrated excellent linearity between Ct values and log_10_(copy number), with R^2^ values of 0.998 for LSDV and 0.9997 for GTPV (Figure 2). The amplification efficiencies were 106.8% for LSDV and 107.6% for GTPV, indicating efficient amplification over the tested concentration range. Slightly elevated efficiencies may be attributable to plasmid quantification or dilution-associated variation.

### 3.3. Analytical Specificity

To evaluate the analytical specificity of the duplex assay, nucleic acid templates from LSDV, GTPV, and a panel of non-target viral and bacterial pathogens were tested under the optimized duplex TaqMan-MGB qPCR conditions. Only the LSDV- and GTPV-positive controls generated target-specific amplification, whereas no amplification was observed for any non-target pathogen or the no-template control (NTC), demonstrating the high analytical specificity of the assay. Detailed specificity results are provided in Supplementary Table S1.

### 3.4. Analytical Sensitivity

**Figure 3:**
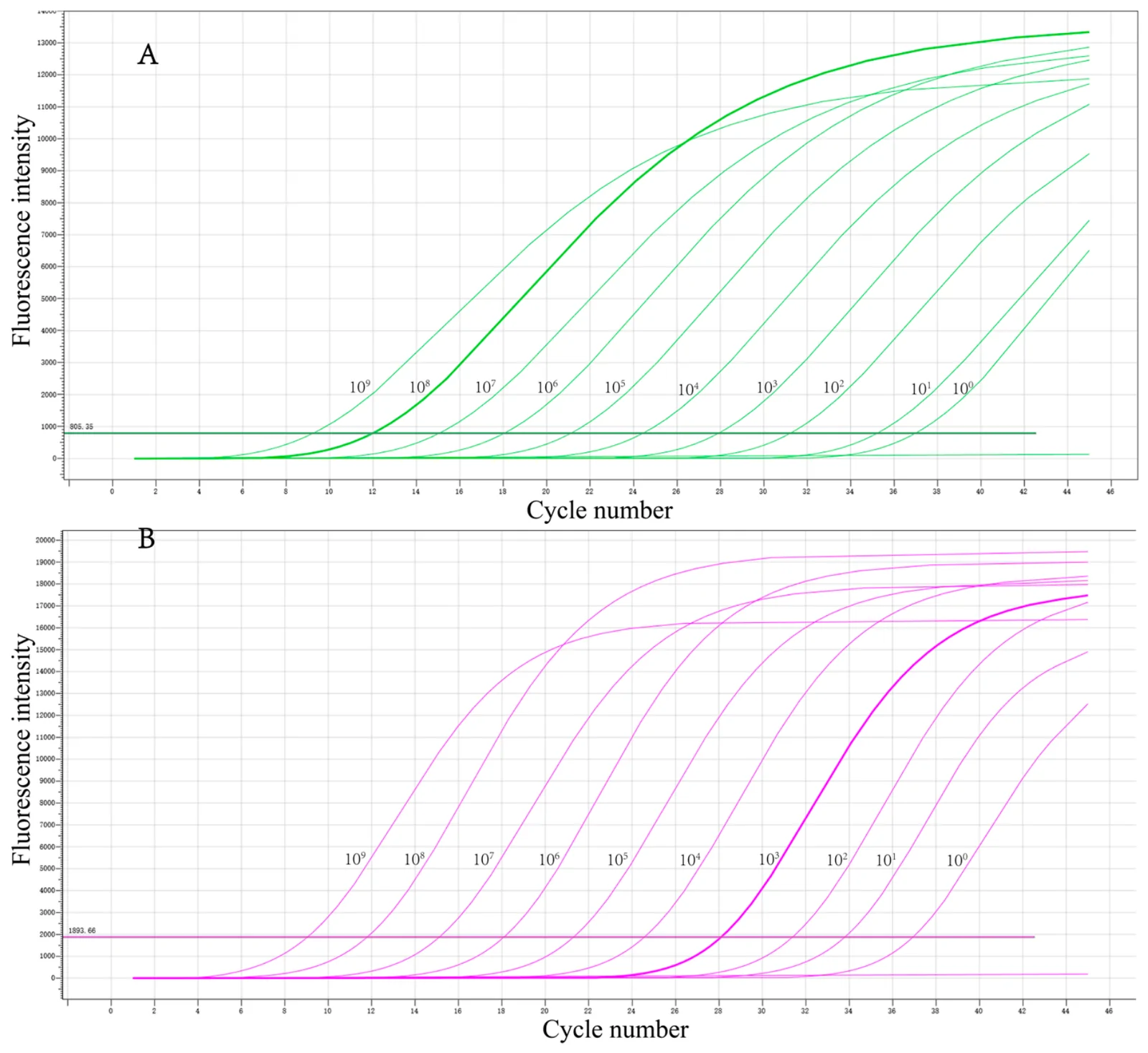
Analytical sensitivity of the Duplex TaqMan-MGB qPCR assay. (**A**) Amplification curves of LSDV obtained from ten-fold serial dilutions of plasmid DNA (1 × 10^9^–1 × 10^0^ copies/μL). (**B**) Amplification curves of GTPV under the same dilution conditions.

Ten-fold serial dilutions of mixed plasmid DNA—pUC57-LSDV and pUC57-GTPV (ranging from 1.0 × 10^9^ to 1.0 × 10^0^ copies/μL)—were amplified using the optimized duplex TaqMan-MGB qPCR assay targeting Chinese circulating LSDV and GTPV. Both targets yielded clear, sigmoidal amplification curves across the dilution series. The assay demonstrated limits of detection (LOD) of 1.0 × 10^1^ copies/μL for GTPV and 1.0 × 10^1^ copies/μL for LSDV (Chinese circulating strain), confirming its high analytical sensitivity (Figure 3).

### 3.5. Reproducibility

Ten-fold serial dilutions of mixed plasmid DNA—pUC57-LSDV and pUC57-GTPV (ranging from 1 × 10^6^ to 1 × 10^4^ copies/μL)—were amplified using the optimized duplex TaqMan-MGB qPCR assay targeting Chinese circulating LSDV and GTPV. As summarized in Table 5, the duplex assay demonstrated excellent reproducibility: intra-assay coefficients of variation (CVs) ranged from 0.38% to 0.66%, whereas inter-assay CVs ranged from 0.10% to 0.81%. Detailed data is presented in Table 5.

### 3.6. Clinical Specimen Testing

A total of 175 yak-derived field specimens from the Qinghai–Tibet Plateau region (Tibet/Qinghai/ Sichuan/Gansu) were analysed, of which 16 and 36 were positive for LSDV and GTPV, respectively. Four dual-positive specimens were detected. When stratified by sampling period, all 16 LSDV-positive specimens were collected during 2019–2024. In contrast, GTPV-positive specimens were detected both before 2019 (3/102) and during 2019–2024 (33/73). To further verify the assay, nucleic acids extracted from five batches of the attenuated GTPV vaccine strain AV41 were tested positive for GTPV nucleic acid across all replicates, confirming the reliable qualitative detection of the vaccine strain. In contrast, the LSDV Neethling strain yielded no amplification, consistent with the intended lineage-selective design of the *GPCR*-targeting system. This system specifically recognizes Chinese epidemic strains (distinguished by variations in the *GPCR* locus) and does not detect non-target lineages such as the Neethling strain, demonstrating the capability to differentiate Chinese epidemic strains from the Neethling strain. Collectively, these findings support the high specificity, sensitivity and practical utility of the established duplex TaqMan-MGB qPCR assay for clinical detection of LSDV and GTPV.

## 4. Discussion

Since the first report in 2019, LSD in China has been characterized by sporadic, multifocal outbreaks with inter-regional spread, and official notifications now cover multiple provinces and regions nationwide [6,7,8,9,10,11]. In October 2021, the first case was identified in yaks. The spread of LSD has resulted in nodular lesions of the skin and mucous membranes accompanied by fever in yaks, leading to high mortality [8]. Secondary bacterial infections can further aggravate the clinical course and cause death [16]. Collectively, these effects have profoundly compromised the health, productivity, and sustainability of yak husbandry on the Qinghai–Tibet Plateau.

GTPV and sheeppox virus (SPPV), together with LSDV, belong to the genus *Capripoxvirus* and are closely related at both the genomic and antigenic levels. This similarity has led several countries to deploy heterologous live-attenuated GTPV or SPPV vaccines for emergency LSD control when homologous LSDV vaccines are unavailable, although protective performance can vary across vaccine strains and settings [17]. Importantly, live capripox vaccination may be followed by transient post-vaccination reactions (e.g., fever and skin nodules/lesions) that resemble mild natural LSD (“Neethling response”), and vaccine virus may be detectable in such lesions, complicating clinical assessment during outbreaks [18]. Moreover, because *Capripoxvirus* are serologically indistinguishable, routine serological assays cannot differentiate antibodies induced by LSDV infection from those elicited by GTPV/SPPV vaccination [17]. In China, where live-attenuated goatpox vaccination has been applied during LSD outbreaks and where vaccine-recombinant LSDV strains have emerged as a distinct circulating subclade, a lineage-specific molecular approach is needed to discriminate field infection from vaccine-related signals and to distinguish Chinese circulating LSDV strains from non-domestic lineages and from GTPV [13]. During LSD outbreaks in China, live-attenuated goatpox vaccination has been implemented, while a vaccine-recombinant LSDV subclade has also emerged. Therefore, a diagnostic method is needed to discriminate Chinese circulating LSDV strains from the GTPV vaccine strain. To address this gap, we developed a TaqMan-MGB probe-based duplex qPCR assay. Compared with conventional hydrolysis probes, MGB probes provide higher sensitivity and enhanced mismatch discrimination, enabling single-nucleotide resolution and thus more precise differentiation between GTPV and regionally distinct LSDV strains. Relevant GTPV and LSDV sequences available in GenBank were aligned using MEGA, and MGB probes were designed based on the observed sequence polymorphisms. At the *RPO30* gene regions, an MGB probe was designed to detect the G→A polymorphism distinguishing GTPV from LSDV. At the *GPCR* gene regions, primers and an MGB probe were designed to differentiate Chinese circulating LSDV strains from foreign LSDV strains and from GTPV by recognizing C/T and C/A substitutions. After optimization of annealing temperature, primer concentrations, and probe concentrations, we established a duplex TaqMan-MGB qPCR for the simultaneous detection of GTPV and Chinese circulating LSDV strains. The assay demonstrated high specificity for both targets and excellent precision, with intra- and inter-assay coefficients of variation (CVs) below 2%. This method allows practical clinical detection of the single-nucleotide polymorphisms (SNPs; C/T and C/A) that distinguish Chinese circulating LSDV strains from foreign isolates while concurrently identifying GTPV, thereby streamlining diagnostic workflows.

Wu et al. developed a TaqMan real-time qPCR assay for LSDV, which exhibited a limit of detection (LOD) of 15 copies/μL and an overall agreement of 65.95% with the World Organisation for Animal Health (WOAH) reference method [19]. Compared with the assay reported by Wu et al., the present duplex assay achieved a lower analytical detection limit and simultaneously detected GTPV in a single reaction. Yao et al. subsequently developed a TaqMan real-time qPCR assay to investigate the prevalence of LSDV in Yunnan Province, achieving an LOD of 4.83 copies/μL [20]. Although their assay showed excellent analytical sensitivity, it was designed for LSDV detection only and did not differentiate GTPV from Chinese epidemic LSDV strains. Yu et al. established a duplex TaqMan real-time qPCR assay for the simultaneous detection of LSDV and GTPV, reporting LODs of 3.37 × 10^1^ copies/μL for both targets and 100% positive concordance with clinical samples [21]. Like the assay developed by Yu et al., the present assay enables simultaneous duplex detection of LSDV and GTPV. However, by incorporating MGB probes targeting lineage-specific polymorphisms in the *GPCR* gene, it further differentiates Chinese epidemic LSDV strains from foreign lineages. Nie et al. developed a real-time qPCR assay for LSDV with an LOD of 4.6 copies/μL; however, in selecting target regions, they did not compare sequences against the recombinant vaccine-like strain LSDV/Russia/Saratov/2017 (GenBank accession no. MH646674), which may result in missed detections [22]. Overall, compared with previously reported qPCR assays, the present duplex TaqMan-MGB assay combines simultaneous detection of GTPV and Chinese epidemic LSDV strains with lineage-specific discrimination in a single reaction.

This study has several limitations. First, specificity against SPPV was assessed in silico but not experimentally validated due to limited access to SPPV-positive material. Second, no endogenous internal amplification control was included in the assay. Therefore, false-negative results caused by PCR inhibition or poor DNA quality cannot be completely excluded. Third, not all clinical positive samples were confirmed by sequencing or an independent reference assay. Fourth, the lineage-selective design of the *GPCR* target means that the assay is intended for Chinese circulating LSDV strains rather than universal detection of all LSDV lineages. Accordingly, the present study represents a preliminary analytical validation rather than a complete diagnostic validation. Further diagnostic validation using well-characterized reference materials and larger independent clinical sample sets will be required before routine diagnostic application. Future studies should expand validation using broader panels of *Capripoxvirus* isolates, incorporate an internal amplification control, and include additional field samples from diverse geographic regions.

## 5. Conclusions

In conclusion, we developed a highly sensitive and specific duplex TaqMan-MGB qPCR assay to simultaneously detect GTPV and Chinese circulating LSDV strains. Importantly, it differentiates Chinese circulating LSDV strains from foreign lineages and readily distinguishes LSDV from GTPV, addressing key limitations of existing molecular diagnostics. Subject to further validation, this duplex TaqMan-MGB qPCR assay could provide a useful tool for early diagnosis, rapid large-scale screening, and epidemiological surveillance.

## Figures and Tables

**Table 1:** *Capripoxvirus* sequences used for primer and probe design.

Virus	Accession No.	Isolate	Sequence Coordinates (bp)
GTPV	MN072621	Vietnam	27,767–110,089
GTPV	MN072622	Turkey	27,555–109,891
GTPV	MN072623	Oman	27,709–110,046
GTPV	MN072625	Yemen	27,808–110,147
SPPV	MN072626	Abu Gharib	26,980–109,304
SPPV	MN072628	Nigeria	27,562–109,915
SPPV	ON961656	Moscow	27,391–111,887
SPPV	MN072630	Saudi Arabia	27,667–111,016
LSDV	MN072619	Kenya	6751–7896
LSDV	KY829023	Evros/GR/15	6923–8056
LSDV	AF409137	Neethling Warmbaths LW	6978–8111
LSDV	MW656253	LSDV/280-KZN/RSA/2018	6750–7883
LSDV	NC_003027	NI-2490	6973–8118
LSDV	OM793607	LSDV_Fourie-FS_RSA_1959	6855–8000
LSDV	KX764644	Neethling-Herbivac vaccine	6983–8128
LSDV	KX764645	Neethling-LSD vaccine-OBP	6960–8105
LSDV	KX764643	SIS-Lumpyvax vaccine	6932–8077
LSDV	MW435866	SA-Neethling	7119–8264
LSDV	OM793608	LSDV_Neethling-WC_RSA_1957	6855–8000
LSDV	OM793609	LSDV_Vaccine_LW-1959_1988	6855–8000
LSDV	OM530217	LSDV/Russia/Saratov/2019	6747–7892
LSDV	MH646674	LSDV/Russia/Saratov/2017	7082–8227
LSDV	MG972412	Cro2016	6899–8044
LSDV	MZ577074	20L43_Ly-Quoc/VNM/20	7060–8205
LSDV	MW732649	LSDV/HongKong/2020	6997–8142
LSDV	OM105589	LSDV/China/XJ01/2019	7098–8243
LSDV	MW355944	China/GD01/2020	7083–8228
LSDV	OP985536	LSDV/MZGD/2020/China	6897–8042
LSDV	OP508345	China/Xinjiang/Cattle/Aug-2019	7021–8166
LSDV	MZ577073	20L42_Quyet-Thang/VNM/20	7060–8205
LSDV	MK441838	Herbivac LS batch 008	6854–7999
LSDV	OP654649	LSDV/China/SiC/2021	6763–7908
LSDV	OM984485	LSDV XJ201901	7084–8229

**Table 2:** Primer and probe sequences.

Target (Gene)	Primers/Probe	Nucleotide Sequence (5′-3′)	Position (bp)	Product Size
GTPV (*RPO30*)	Forward	AGTAATGGTAGGATAGTCGCAAATGA	61,134–61,159	87 bp
	Reverse	GTCAGATATAAACCCGGCAAGTG	61,198–61,220	
	Probe	VIC-AGTTGCAATGCGTAGTATG-MGB	61,178–61,196	
LSDV (*GPCR*)	Forward	TGTTCAGTATTGTTTTTACTCCCATTTAG	6980–7008	140 bp
	Reverse	TGACATAGAGACACAATTTCAGCTACA	6869–6895	
	Probe	FAM-ATGTACGGCATTACGA-MGB	6916–6931	

Note: The primer and probe sequences for LSDV were designed based on the reference sequence LSDV isolate China/SiC/2021 (GenBank accession OP654649.1), whereas those for GTPV were designed based on goatpox virus isolate Turkey, complete genome (GenBank accession MN072622.1).

**Table 3:** Optimization of primer concentrations for the Duplex TaqMan-MGB qPCR assay.

Group	Probe Volume (µL/10 µM)		Ct Values	
	LSDV	GTPV	LSDV	GTPV
1	0.2	0.2	19.33	18.62
2	0.3	0.2	18.61	18.56
3	0.4	0.2	18.63	18.69
4	0.2	0.3	19.02	18.64
5	0.3	0.3	18.88	18.43
6	0.4	0.3	18.59	18.42
7	0.2	0.4	18.65	20.41
8	0.3	0.4	18.46	19.54
9	0.4	0.4	18.41	19.21

**Table 4:** Optimal Probe Concentration Results of the Duplex TaqMan-MGB qPCR assay.

Group	Probe Volume (µL/10 µM)		Ct Values	
	LSDV	GTPV	LSDV	GTPV
1	0.2	0.2	17.83	18.28
2	0.3	0.2	17.42	18.07
3	0.4	0.2	17.37	18.05
4	0.2	0.3	16.98	17.80
5	0.3	0.3	17.44	17.70
6	0.4	0.3	17.23	17.90
7	0.2	0.4	17.79	17.68
8	0.3	0.4	17.36	17.52
9	0.4	0.4	17.23	17.40

**Table 5:** Intra- and inter-assay reproducibility of the Duplex TaqMan-MGB qPCR assay for LSDV and GTPV (*n* = 3 independent experiments, each tested in triplicate).

Plasmid	Concentration (Copies/μL)	Intra-Assay Ct (Mean ± SD)	CV (%)	Inter-Assay Ct (Mean ± SD)	CV (%)
LSDV	1 × 10^6^	17.37 ± 0.07	0.38%	17.41 ± 0.13	0.76%
	1 × 10^5^	20.87 ± 0.14	0.66%	20.81 ± 0.12	0.56%
	1 × 10^4^	24.11 ± 0.14	0.60%	24.12 ± 0.16	0.65%
GTPV	1 × 10^6^	17.62 ± 0.09	0.49%	17.47 ± 0.14	0.81%
	1 × 10^5^	21.08 ± 0.10	0.49%	20.95 ± 0.06	0.29%
	1 × 10^4^	24.47 ± 0.15	0.61%	24.47 ± 0.03	0.10%

## Data Availability

The raw quantitative data generated and analyzed during this study are available from the corresponding author upon reasonable request. All processed data supporting the findings of this study are included in the article and its Supplementary Materials.

## Supplementary Materials

The following supporting information can be downloaded at https://www.mdpi.com/article/10.3390/ani16162561/s1. Table S1: Analytical specificity of the duplex TaqMan-MGB qPCR assay; Table S2: Ct values and interpretation of all clinical specimens tested by the duplex TaqMan-MGB qPCR assay.
