# Supplementary material for: Development of a Duplex TaqMan-MGB qPCR Assay for Differential Detection of Chinese Epidemic Lumpy Skin Disease Virus Strains and Goatpox Virus
Source: Animals (Basel). 2026 Aug 17;16(16):2561. doi: 10.3390/ani16162561 (PMC13508980; doi:10.3390/ani16162561)
Supplement: Supplementary file 1 [file animals-16-02561-s001.zip › animals-4460450-supplementary.pdf]

Supplementary Table S1. Analytical specificity of the duplex TaqMan-MGB qPCR assay.

Template	FAM channel (Ct)	VIC channel (Ct)	Interpretation
LSDV/China/SiC/2021	17.63	ND	Positive in FAM only
GTPV vaccine strain AV41	ND	18.24	Positive in VIC only
SPPV	ND	ND	Negative
Orf virus	ND	ND	Negative
PPRV	ND	ND	Negative
BVDV	ND	ND	Negative
BCoV	ND	ND	Negative
BRV	ND	ND	Negative
BHV-1	ND	ND	Negative
E. coli	ND	ND	Negative
S. aureus	ND	ND	Negative
Salmonella	ND	ND	Negative
No-template control (NTC)	ND	ND	Negative

Supplementary Table S2. Ct values and interpretation of all clinical specimens tested by the duplex TaqMan-MGB qPCR assay.

Sample No.	FAM channel (Ct value)	VIC channel(Ct value)	Interpretation
1	38.00	37.31	Negative
2	38.33	39.95	Negative
3	41.10	39.79	Negative
4	38.04	40.01	Negative
5	41.76	37.94	Negative
6	36.71	37.03	Negative
7	38.46	41.05	Negative
8	40.14	41.11	Negative
9	37.56	40.68	Negative
10	41.68	40.08	Negative
11	37.05	39.46	Negative
12	37.77	36.05	Negative
13	41.00	40.40	Negative
14	38.14	38.62	Negative
15	41.84	42.92	Negative

16	40.70	38.89	Negative
17	38.49	36.91	Negative
18	41.71	42.70	Negative
19	36.86	40.96	Negative
20	41.30	39.11	Negative
21	41.10	39.68	Negative
22	36.59	36.64	Negative
23	41.65	38.67	Negative
24	37.65	40.20	Negative
25	37.57	38.86	Negative
26	41.31	41.49	Negative
27	38.03	37.06	Negative
28	40.57	40.02	Negative
29	39.51	37.23	Negative
30	39.35	41.66	Negative
31	41.67	36.95	Negative
32	38.40	36.61	Negative
33	38.38	42.01	Negative
34	38.30	40.60	Negative
35	39.94	36.41	Negative
36	41.41	36.65	Negative
37	39.24	37.50	Negative
38	40.49	37.99	Negative
39	37.18	41.58	Negative
40	38.21	36.14	Negative
41	38.08	42.87	Negative
42	36.95	36.49	Negative
43	37.68	37.58	Negative
44	37.17	41.17	Negative
45	38.63	37.82	Negative
46	41.17	42.68	Negative
47	41.40	36.63	Negative
48	40.20	36.31	Negative
49	39.65	40.75	Negative
50	41.72	40.20	Negative
51	36.08	41.07	Negative
52	40.72	38.70	Negative
53	36.99	41.10	Negative
54	40.78	36.67	Negative
55	38.82	40.55	Negative
56	37.63	37.53	Negative
57	38.58	38.52	Negative
58	37.69	39.75	Negative
59	40.04	41.21	Negative

60	38.32	40.69	Negative
61	41.09	38.40	Negative
62	41.81	41.06	Negative
63	38.85	36.29	Negative
64	40.58	39.32	Negative
65	41.13	41.32	Negative
66	39.53	40.79	Negative
67	37.26	42.56	Negative
68	39.65	37.68	Negative
69	40.61	36.07	Negative
70	41.65	36.91	Negative
71	41.75	36.65	Negative
72	40.08	42.53	Negative
73	36.33	41.89	Negative
74	38.03	38.36	Negative
75	37.57	37.25	Negative
76	40.07	40.15	Negative
77	38.16	36.54	Negative
78	37.71	39.84	Negative
79	37.00	39.58	Negative
80	39.90	40.61	Negative
81	39.20	36.38	Negative
82	36.08	37.58	Negative
83	36.75	40.47	Negative
84	40.65	42.37	Negative
85	41.10	36.01	Negative
86	40.66	41.98	Negative
87	39.70	39.64	Negative
88	37.30	39.93	Negative
89	38.40	41.12	Negative
90	41.29	37.40	Negative
91	40.67	37.53	Negative
92	36.01	41.31	Negative
93	37.99	36.14	Negative
94	37.35	37.81	Negative
95	36.26	39.72	Negative
96	36.26	42.02	Negative
97	41.57	37.10	Negative
98	37.35	38.48	Negative
99	37.68	39.50	Negative
100	37.57	39.01	Negative
101	36.50	36.29	Negative
102	38.02	40.60	Negative
103	41.97	38.26	Negative

104	40.50	42.72	Negative
105	36.85	36.02	Negative
106	37.97	40.29	Negative
107	37.26	36.19	Negative
108	37.21	36.97	Negative
109	41.50	36.40	Negative
110	37.01	37.46	Negative
111	39.98	39.61	Negative
112	40.91	36.74	Negative
113	40.55	36.94	Negative
114	38.23	38.64	Negative
115	40.71	38.18	Negative
116	36.73	38.89	Negative
117	39.42	37.00	Negative
118	37.48	37.64	Negative
119	41.69	39.95	Negative
120	39.94	40.19	Negative
121	38.19	41.57	Negative
122	41.75	40.35	Negative
123	41.35	36.06	Negative
124	39.54	36.58	Negative
125	39.12	36.15	Negative
126	37.99	40.39	Negative
127	37.41	40.44	Negative
128	38.13	27.71	GTPV-positive
129	39.28	30.38	GTPV-positive
130	40.76	29.65	GTPV-positive
131	39.74	29.05	GTPV-positive
132	37.05	27.22	GTPV-positive
133	38.40	29.36	GTPV-positive
134	37.52	25.41	GTPV-positive
135	38.44	29.07	GTPV-positive
136	39.29	30.34	GTPV-positive
137	39.07	32.15	GTPV-positive
138	39.33	31.11	GTPV-positive
139	39.07	30.99	GTPV-positive
140	38.53	26.67	GTPV-positive
141	39.06	27.50	GTPV-positive
142	36.57	26.65	GTPV-positive
143	38.30	32.53	GTPV-positive
144	41.32	28.79	GTPV-positive
145	39.87	26.31	GTPV-positive
146	37.56	31.83	GTPV-positive
147	36.23	32.88	GTPV-positive

148	37.59	28.53	GTPV-positive
149	38.08	29.84	GTPV-positive
150	41.24	31.88	GTPV-positive
151	38.41	20.94	GTPV-positive
152	41.31	25.16	GTPV-positive
153	41.57	23.26	GTPV-positive
154	36.62	26.25	GTPV-positive
155	39.38	30.88	GTPV-positive
156	38.29	31.73	GTPV-positive
157	40.69	27.60	GTPV-positive
158	36.11	27.48	GTPV-positive
159	36.68	24.55	GTPV-positive
160	32.32	27.40	LSDV/GTPV co-positive
161	21.08	24.48	LSDV/GTPV co-positive
162	23.69	22.01	LSDV/GTPV co-positive
163	33.48	27.09	LSDV/GTPV co-positive
C10	27.76	41.86	LSDV-positive
C15	27.42	41.88	LSDV-positive
C16	28.31	42.39	LSDV-positive
B1	26.69	42.05	LSDV-positive
F2	20.57	40.23	LSDV-positive
F3	22.63	42.34	LSDV-positive
N1	26.97	39.68	LSDV-positive
N3	27.28	41.93	LSDV-positive
N4	26.09	39.66	LSDV-positive
S2	16.72	39.93	LSDV-positive
S3	21.47	39.49	LSDV-positive
S1	17.47	39.81	LSDV-positive
